# A reliable and valid tool to assess the sexual acceptability of contraceptive methods

**DOI:** 10.3389/fpubh.2023.1302675

**Published:** 2024-01-04

**Authors:** Isabel Lahoz-Pascual, Ana Rosa Jurado-Lopez, Raúl Juárez-Vela, Iván Santolalla-Arnedo, Regina Ruiz de Viñaspre-Hernández, Sira Repollés-Lasheras, Susana Tejero Sancho, Miguel Diaz-Vega, Ana Cristina Lou-Mercade, Nicolás Mendoza-Ladrón de Guevara

**Affiliations:** ^1^Department of Obstetrics and Gynecology, Hospital Clínico Universitario Lozano Blesa, Zaragoza, Spain; ^2^Department of Obstetrics and Gynecology, University of Zaragoza, Zaragoza, Spain; ^3^Department of Obstetrics and Gynecology, University of Granada, Granada, Spain; ^4^Department of Nursing, University of La Rioja, Logroño, Spain; ^5^Biomedical Research Center of a Rioja, CIBIR, Logroño, Spain

**Keywords:** questionnaire development, choice of contraceptive method, sexual acceptability, gynecology, obstetrics

## Abstract

**Introduction:**

Adequate identification of the sexual acceptability of contraceptive methods is key for designing health promotion interventions, assessing their impacts, and increasing their effectiveness. This study aimed to develop and validate a questionnaire to explore the preferences of women depending on their epidemiological characteristics and their partner relationships—the *Sexual Acceptability of Contraceptive Methods Questionnaire* [in Spanish, Aceptabilidad Sexual de los Métodos Anticonceptivos (ASMA)].

**Methods:**

Psychometric validation was conducted using Exploratory Factorial Analysis (EFA) and confirmatory factor analysis (CFA). The reliability of the final version of the questionnaire was explored using Cronbach’s alpha and McDonald omega to estimate internal consistency.

**Results:**

A three-factor model was identified. Factor 1 (explaining 28.32% of the model) corresponds to questions concerning the use and placement of the contraceptive and includes 6 items; Factor 2 (explaining 24.23%) corresponds to other factors that affect the relationship such as bleeding and side effects of the contraceptive method and includes 10 items; and Factor 3 (explaining 18.94%) corresponds to the couple relationship and includes 8 items.

**Conclusion and implications:**

The ASMA questionnaire provides a valid and reliable tool for assessing the sexual acceptability of various contraceptive methods. This instrument gathers data that provide information on various aspects of women’s sexuality, health, education, and beliefs, all of which can determine the preference for one contraceptive method over another. Moreover, the tool can help to identify profiles of women who have different preferences when selecting a particular method.

## Introduction

The aim of contraception is to avoid unwanted pregnancies and ensure a satisfactory sexual life free of procreative risks (1). In Spain, the contraceptive method most commonly used by women in their sexual relations is the condom, followed by the pill. It is important to emphasize that there has been no change in the pattern of use since 1997 despite the marketing of many other types of more effective and comfortable contraceptive methods that can be used during sexual intercourse (2). The literature includes studies that analyze the factors associated with the choice of contraceptive in relation to the sexual life of women. These studies highlight variables such as the subjective characteristics of the woman, and the availability and ease of use of the method (3–5).

In addition, the cost associated with unwanted pregnancies is high. The majority of unintended pregnancies (26%) occur in women aged 30–34 years, while an estimated 69% of the total cost burden is attributable to poor adherence to contraceptive methods, with such a cause most likely being avoidable with the intervention of a health professional (6).

Adequate identification of the sexual acceptability of contraceptive methods is key for designing health promotion interventions, assessing their impacts, and increasing their effectiveness. Currently, there are almost no standard instruments in the scientific literature to assess the sexual acceptability of contraceptive methods. Although one tool has been published recently to measure how the contraceptive method affects women’s sexuality, this cannot be used as a means of matching the contraceptive method with women’s sexual preferences (7). Thus, there is a need for instruments that can be used in any context to determine which contraceptive method is preferred by women according to various factors, including their relationships.

This study aimed to develop and validate a Sexual acceptability of Contraceptive Methods questionnaire to explore women’s preferences according to their epidemiological characteristics and their partner relationships. This instrument is called the *Sexual Acceptability of Contraceptive Methods Questionnaire* [in Spanish, Aceptabilidad Sexual de los Métodos Anticonceptivos (ASMA)].

## Methods

The study methodology included questionnaire development and validation.

### Development stage

The development of the tool began with a conceptualization phase and was followed by the design and pilot testing of the questionnaire.

#### Conceptualization

he questionnaire conceptualization and construct (dimension) definition were carried out considering the following: (1) the aim of the tool; (2) a bibliographic review on the topic using the CINAHL, PubMed, Psychinfo, and SciELO databases, combining the search terms “(contraception OR contraceptive OR contraceptive device OR contraceptive devices) AND (pleasure OR libido OR “sexual function” OR “sexual functioning” OR sexuality NOT [“sexual behavior” OR “sexual health”]),” and institutional web pages (e.g., World Health Organization); (3) a qualitative study with 6 experts on contraception and/or sexuality issues and content analysis; and (4) the research team’s experience. The experts have been selected from among medical professionals with training in contraception and sexology and with research experience.

#### Questionnaire design

The questionnaire was designed to include nominal scale items to collect sociodemographic/epidemiological information and Likert or nominal scales to assess each dimension. The item sequence was determined considering the construct criteria, order, and specificity. The questionnaire was edited for length and format.

Inclusion criteria were: Female between 16 and 50 years, currently having sex. Adequate command of oral and written Spanish and overt desire to use contraceptive measures. Data collection took place in gynecology consultations during a full year at the Hospital Clínico Universitario Lozano Blesa in Zaragoza (Spain).

#### Pilot

To assess the acceptability and content validity of the questionnaire, the first draft (66 items, 28 sociodemographic/epidemiological and 38 sexual/contraceptive-related questions) was reviewed independently by seven key experts. A copy of the first draft of the questionnaire was sent to the experts via email. The purpose of this revision was to eliminate questions based on their relevance for achieving the objectives of the study (items that more than 70% of the experts believed to be unsuitable/irrelevant for the study were eliminated), and to modify the wording of some items to facilitate understanding by any person regardless of their cultural, social, and economic level.

Then, a second draft of the questionnaire (55 items: 28 sociodemographic/epidemiological and 27 sexual/contraceptive-related questions) was distributed to a convenience sample of 216 women. A descriptive analysis was then conducted to explore the frequency distributions and ceiling and roof effects (>70% of the responses grouped under the extreme options) and reliability, using Cronbach’s alpha to estimate internal consistency. In all instances, the participants were informed about the study by one of the researchers and were also provided with written information. In the initial stages, written consent was obtained from all participants.

#### Psychometric validation stage

After the pilot study, a psychometric validation of the first version of the questionnaire (52 items: 28 sociodemographic/epidemiological and 24 sexual/contraceptive-related questions) was carried out (Supplementary Appendix 1).

Two recommendations were considered to estimate the sample size at this stage, that is, 8–10 participants were needed for each item in the questionnaire and 192–240 participants were required to obtain a “very good” sample for conducting factor analysis (8); at least 216 were needed for EFA while a subsample of 83 participants were required to analyze test–retest reliability.

The Doornik-Hansen test for normality analysis was conducted on the data collected, and a descriptive analysis was performed to explore each item’s acceptability and to identify the ceiling and roof effects. Exploratory factor analysis (EFA) (9, 10) and confirmatory factor analysis (CFA) were carried out in that order to explore the construct validity of the questionnaire (11). Sampling adequacy was assessed using the Kaiser-Meyer-Olkin (KMO) test and the suitability of the data for factor analysis was confirmed using Bartlett’s test of sphericity. Structural equation modeling (SEM) was applied, and the maximum likelihood method was used for estimation. The normed fit index (NFI), root mean square error of approximation (RMSEA), chi square test, and comparative fit index (CFI) were used to test the goodness-of-fit (12). Correlations between the dimensions were explored through the Spearman correlation coefficient.

The reliability of the final version of the questionnaire was examined through Cronbach’s alpha and McDonald omega for internal consistency.

All of the quantitative analyses were conducted using STATA statistical software (Stata Corp, College Station, Texas, United States).

This study was evaluated by the local ethics research committee (code: 22/2020). Written information about the study was provided to participants, and their consent to participate was assumed when they returned the completed questionnaire.

## Results

### Descriptive analysis

Table 1 reports the sociodemographic characteristics of the 216 participants. The mean age was 33.98 years (dt:7.74) with a BMI classified as normal weight. Of the sample, 21.30% had chronic diseases, while it is also worth mentioning that 15.74% had induced abortion.

**Table 1:** Sociodemographic characteristics of the sample.

Variable	Frequency (percentage)	
Age (Mean, SD)	33.98 (7.74)	
IMC (Mean, SD)	24.46 (5.34)	
Chronic disease	46 (21.30%)	
Vaginal deliveries	136 (62.96%)	
Number of deliveries	1.18 (1.26)	
Cesarean	32 (14.81%)	
Spontaneous abortions	26 (12.04%)	
Induced abortions	34 (15.74%)	
Gynecological history	30 (13.89%)	
Chronic drugs	47 (21.76%)	
Habitual intake of intoxicants	64 (29.63%)	
Personal socioeconomic level		
	No income	44 (20.37%)
	0 to 12.450 (€)	95 (43.98%)
	12.450 a 20.200 (€)	51 (23.61%)
	20.200 a 35.200 (€)	22 (10.19%)
	35.200 a 60.000 (€)	3 (1.39%)
	>60.000 (€)	1 (0.46%)
Family socioeconomic level		
	No income	4 (1.85%)
	0 to 12.450 (€)	51 (23.61%)
	12.450 a 20.200 (€)	74 (34.26%)
	20.200 a 35.200 (€)	59 (27.31%)
	35.200 a 60.000 (€)	24 (11.11%)
	>60.000 (€)	4 (1.85%)
Employment status		
	Self-employed	21 (9.72%)
	Employment as an employee	22 (10.19%)
	Unemployed	126 (58.33%)
	Unpaid household work	21 (9.72%)
	Student	25 (11.57%)
	Disabled	1 (0.46%)
Education level		
	No studies	10 (4.63%)
	Primary studies	45 (20.83%)
	Secondary education	104 (48.15%)
	University	57 (26.39%)
Type of education according to financing		
	Did not attend school	7 (3.24%)
	Public	153 (70.83%)
	Public/privately funded	55 (25.46%)
	Private	1 (0.46%)
Type of school attended according to religious education		
	Did not attend school	66 (30.56%)
	Lay	149 (68.98%)
	Religious	1 (0.46%)
Religion		
	Agnostic	19 (8.80%)
	Atheist	32 (14.81%)
	Muslim	14 (6.48%)
	Practicing Catholic	30 (13.89%)
	Non-practicing Catholic	84 (38.89%)
	Evangelist	13 (6.02%)
	Jehovah’s witness	3 (1.39%)
	Other	21 (9.72%)
Origin/ethnicity		
	North American	3 (1.39%)
	South American	35 (16.20%)
	Spanish	131 (60.65%)
	European	19 (8.80%)
	Eastern countries	8 (3.70%)
	Maghreb	10 (4.63%)
	Sub-Saharan African	7 (3.24%)
	Gypsy	3 (1.39%)

Moreover, 43.98% of the women had an income below 12,450 euros, 34.26% between 12,450 and 20,200 and 27.31% more than 20,200. Regarding employment and education status, 58.33% were unemployed and 48.15% had a high school education, while 70.83% were educated in public schools and 68.98% secular schools.

Table 2 describes the participants’ relationships and sexual preferences. Only 15.28% did not have a steady partner and 13.43% had a steady partner but did not live with them. Concerning the length of time in a couple relationship, 44.91% of the women had been co-habiting with their partner for more than 10 years, while 9.17% had two current sexual partners and 19.91% had had more than one relationship at the same time. A total of 86.57% declared themselves heterosexual and 63.43% of the respondents had sex on a weekly basis.

**Table 2:** Description of the sexual relations and contraceptive methods used by the participants.

Variable	Frequency (percentage)	
Type of partner		
	Living together as a couple	154 (71.29%)
	Steady partner not living together	29 (13.43%)
	No steady partner	33 (15.28%)
Time with partner		
	No steady partner	33 (15.28%)
	Less than 2 years	24 (11.11%)
	2–5 years	28 (12.96%)
	5–10 years	34 (15.74%)
	> 10 years	97 (44.91%)
Length of co-habitation		
	No co-habitation	62 (28.70%)
	Less than 2 years	22 (10.19%)
	2–5 years	24 (11.11%)
	5–10 years	27 (12.50%)
	> 10 years	81 (37.50%)
Number of CURRENT sexual partners		
	0	11 (5.09%)
	1	196 (90.74%)
	2	9 (4.17%)
Sexual relationships at the same time		
	No	173 (80.09%)
	Yes	43 (19.91%)
Caregivers in the home		
	0	71 (32.87%)
	1	143 (66.20%)
	2	2 (0.93%)
Sexual orientation		
	Homosexual	8 (3.70%)
	Bisexual	18 (8.33%)
	Heterosexual	187 (86.57%)
	Other	3 (1.39%)
Sexual activity		
	Daily	17 (7.87%)
	Weekly	137 (63.43%)
	Monthly	29 (13.43%)
	Sporadic	33 (15.28%)
Frequency of contraceptive use		
	Always	134 (62.04%)
	Almost always	36 (16.67%)
	Sometimes	26 (12.04%)
	Never	20 (9.26%)
Reason for change		
	Problems with frequency of use	32 (14.81%)
	Adverse effects	40 (18.52%)
	Problems with sexual relations	38 (17.59%)
	Economic issues	3 (1.39%)
	Effectiveness	48 (22.22%)
	Pregnancy during use	3 (1.39%)
	Other	52 (24.07%)

With respect to contraceptive use, 9.26% never used contraceptives and only 12.04% used them occasionally. The most commonly used contraceptives were the classic combined pill and the gestagen-only pill, used by more than 50% of them. The reason for wanting to change contraceptive methods was the appearance of adverse effects, efficacy, and problems with sexual intercourse.

The sample size for the factor analysis consisted of the 24 sexual/contraceptive-related items. The Kaiser-Meyer-Olkin (KMO) value was 0.7873, considered adequate for the factor analysis, and the Bartlett’s test of sphericity also demonstrated satisfactory suitability of the data for factor analysis (*p* < 0.001). A loading cutoff of >1 was adopted, complying with Kaiser’s rule, and three factors were extracted. This structure accounted for 71.50% of the model variance (see Table 3).

**Table 3:** Exploratory factor analysis showing total variance explained with three factors.

Factor	Eigenvalue	Difference	% factor variance	% total variance
Factor 1	1,2045	0.17395	0.2832	0.2832
Factor 2	1,0305	0.22494	0.2423	0.5255
Factor 3	1,0112	0.01935	0.1894	0.7150

The three-factor structure of the model is described in Table 4, where three conceptual dimensions were identified. Factor 1 (explaining 28.32% of the model) corresponds to questions related to the use and placement of the contraceptive method and includes 6 items; Factor 2 (explaining 24.23%) corresponds to other factors that affect the relationship such as bleeding and side effects of the contraceptive method and includes 10 items; and Factor 3 (explaining 18.94%) corresponds to the couple relationship and includes 8 items.

**Table 4:** Exploratory factor analysis results for the ASMA questionnaire.

Item	Factor 1	Factor 2	Factor 3
p1			0.3803
p2			0.4251
p3			0.4833
p4			0.2099
p5			0.2805
p6			0.2112
p7	0.3627		
p8	0.3185		
p9	0.2365		
p10	0.3385		
p11	0.3548		
p12	0.3412		
p13		0.3989	
p14		0.3245	
p15		0.2186	
p16		0.2218	
p17			0.2253
p18		0.2728	
p19		0.3243	
p20		0.2177	
p21		0.3379	
p22		0.2140	
p23		0.3661	
p24			0.2611

To analyze the internal consistency or homogeneity of the instrument, Cronbach’s alpha and McDonald omega were used for each of the dimensions obtained in the AF. Table 5 shows the values obtained, where we can observe high values close to unity, indicating that each of the factors yielded by the factor analysis is consistent in the sense that its constituent items are stable in this dimension.

**Table 5:** Cronbach’s alpha and McDonald’s omega for factors and total questionnaire.

Factor	Cronbach’s alpha	McDonald’s omega
Factor 1	0.8388	0.8048
Factor 2	0.7501	0.7440
Factor 3	0.6631	0.6157
Total	0.7038	0.7017

Following the EFA, CFA was conducted, revealing a good fit to the model (NFI = 0,741; RMSEA = 0.048; CFI = 0.779), which indicated that the theoretical three-factor structure of this model was adequate.

## Discussion

This is one of the first studies to develop and validate a tool to assess the sexual acceptability of contraceptive methods. The ASMA questionnaire evaluates the acceptability of contraceptive methods considering epidemiological factors as well as lifestyle, socioemotional aspects, educational level, and religious beliefs.

Until now, the scientific literature has only presented a single questionnaire for studying the Contraceptive Sexual Acceptability (CSA scale) (7). These authors developed a tool based on the conceptual model of the sexual acceptability of contraception (5, 13, 14). This recent scale provides a measure that asks women to rate their satisfaction with physical (including sexual functioning, quality, frequency of orgasm, and arousal), psychological (emotional openness or capacity for surrender) and interpersonal domains. The CSA scale was created to identify the acceptability of contraceptives and to measure the changes in sexuality that users attribute to a new contraceptive method. This scale is based on classic questionnaires in the literature (FSFI, NSSS), which evaluate the various aspects of female sexual response during the use of contraceptive methods. In this regard, the ASMA scale can be used in women seeking contraception, allowing the clinician to recommend the most suitable method according to the woman’s sexual preferences and particularities.

Caruso et al. (15) analyzed the impact of hormonal contraception on women’s sexuality, focusing on aspects such as their components, route, and regimen (15). In this regard, the ASMA questionnaire was created to identify key elements of the contraceptive sexual acceptability construct. Another novel aspect of our questionnaire instrument compared with that described by Caruso et al. (15) is that the ASMA questionnaire seeks to evaluate women’s preferences according to epidemiological characteristics, where we include age and sexual orientation and type of partner relationship, along with socioeconomic, educational, and religious factors.

The central aim of the ASMA questionnaire was to identify profiles of factors associated with the different types of contraceptives. Therefore, this instrument is the first to comprehensively assess women’s contraceptive needs with the aim of achieving full satisfaction in their sexual relationships.

All of the items included in the scale are critical for evaluating the sexual acceptability of contraceptives. The questionnaire begins with demographic and clinical questions about the woman’s medical history that may affect her sexual relations or preference for a particular type of contraceptive. This is followed by personal and family economic questions, as well as the type of relationship and cohabitation with the partner. All these questions are of great relevance for establishing a woman’s profile for a given type of contraceptive method. In refining this part of the scale, statistically informed decisions were made based on the results of the analysis. The second part of the scale consists of questions related to the reality of their sexual lives, enquiring about aspects that can be influenced by the various contraceptive methods. The responses can then be used as a basis for providing contraceptive counseling.

Although the questionnaire is a helpful tool for contraceptive counseling and prescription, we must always bear in mind that during the consultation the user might also communicate other individual variables not reflected in the questionnaire, and such information could also be decisive in the choice of contraceptive method.

The internal theoretical structure of the initial questionnaire was slightly modified, maintaining 3 factors but slightly changing the way of grouping the items.

However, all these constructs are theoretically consistent and present good internal consistency and reliability. In future studies, it will be useful to explore the correlations between factors.

### Limitations and strengths

The lack of similar instruments for assessing the acceptability of contraceptive methods did not allow us to assess criterion validity against a “gold standard” (9). However, having a frame of reference defined by health professionals for the subject under analysis along with a content and appearance validation of the questionnaire conducted by experts is one of the strengths in the design of this preliminary instrument. Although psychometric validation analysis revealed that the ASMA questionnaire is valid and reliable, additional validations in different populations could contribute to its improvement (10). Given that a high percentage of the sample were non-Spanish women, the results of the analysis suggest good cultural adaptability.

### Future usefulness of the tool

The developed instrument could help health professionals in routine clinical practice to identify the best contraceptive method for a woman who attends the clinic seeking information and help in this regard, identifying the method that best suits the profile of each individual.

In conclusion, the ASMA questionnaire is a valid and reliable tool for assessing the sexual acceptability of various contraceptive methods. The ASMA questionnaire collects data and provides information on different factors concerning women’s sexuality, health, education, and beliefs — factors that can determine the preference for one contraceptive method over another. It can thus be used to identify profiles of women with different preferences, helping them to choose the most suitable method.

## Data availability statement

The datasets presented in this article are not readily available because of data protection law. Requests to access the datasets should be directed to isalahoz@yahoo.com.

## Ethics statement

Written informed consent to participate in this study was provided by the participants. The studies involving human participants were reviewed and approved by the Aragon Research Ethics Committee-CEICA with protocol number PI20/488.

## Author contributions

IL-P: Conceptualization, Investigation, Software, Writing – original draft, Writing – review & editing. AJ-L: Data curation, Methodology, Supervision, Writing – original draft. RJ-V: Funding acquisition, Resources, Visualization, Writing – original draft. IS-A: Funding acquisition, Resources, Visualization, Writing – original draft. RR: Funding acquisition, Resources, Visualization, Writing – original draft. SR-L: Formal analysis, Project administration, Writing – original draft. ST: Methodology, Project administration, Supervision, Validation, Writing – original draft. MD-V: Data curation, Formal analysis, Investigation, Software, Writing – original draft. AL-M: Investigation, Methodology, Resources, Writing – original draft. NM-L: Conceptualization, Formal analysis, Methodology, Supervision, Writing – original draft, Writing – review & editing.
