# Supplementary material for: A reliable and valid tool to assess the sexual acceptability of contraceptive methods
Source: Front Public Health. 2024 Jan 4;11:1302675. doi: 10.3389/fpubh.2023.1302675 (PMC10798038; doi:10.3389/fpubh.2023.1302675)
Supplement: Supplementary file 1 [file Table_1.DOCX]

Appendix 1: Questionnaire

EPIDEMIOLOGICAL DATA

1. Age:
2. Weight:
3. Height:
4. BMI:

Low Weight: <18.5

Normal: 18.5-24.99

Overweight: 25-29.99

Obesity:>30

Morbid Obesity:>35%.

1. Chronic illnesses: YES NO. Specify if yes.

Diabetes

HTA

Metabolic syndrome

Cardiovascular diseases

Psychiatric/Psychological illness: (e.g., stress, chronic fatigue, fibromyalgia, depression) Epilepsy

Neuromuscular disease

Oncological disease

Other.

1. Number of pregnancies:

Number of vaginal deliveries ( )

Number of caesarean sections ( )

Number of miscarriages ( )

Number of induced abortions ( ).

1. Gynecological history:
   - YES
   - NO.
2. Specify if yes...: malformations/surgery.../diseases: endometriosis/myoma/chronic pelvic pain/sexually transmitted diseases/other).
3. Chronic drugs:
   - YES
   - NO.
4. Please specify if yes.

Cardiovascular (anti-HTA-diuretics-diuretics-digoxin-amiodarone)

Antidiabetics

Hypercholesteremics

Antidepressants

Psychotropic drugs

ACHO

Antiepileptics

Antihistamines (cimetidine/ranitidine)

Antineoplastics

Anti-androgens

Corticosteroids

GnRH agonists

Immunosuppressants

Ketoconazole

Naproxen

Levodopa

Protease inhibitors

Other

1. Regular intake of intoxicants:
   - YES
   - NO.
2. Please specify if yes.

Tobacco

Alcohol

Cannabis

Cocaine

Others.

1. Annual personal socio-economic level:

No personal income

0 to 12,450

12,450 to 20,200

20,200 to 35,200

35,200 to 60,000

>60.000

1. Annual family socio-economic level:

No personal income

0 to 12,450

12,450 to 20,200

20,200 to 35,200

35,200 to 60,000

>60.000

1. Employment status:

Self-employed

Employed

Unemployed

Unpaid work at home

Student

Pensioner

Incapacity for work

1. Education level:

No education (or incomplete primary education)

Primary education

Secondary education (baccalaureate)

University studies

1. Type of school attended according to school funding

Did not attend school

Private

Private/public funding

Public

1. Type of school attended according to religious education

Did not attend school

Lay

Religious

1. Religion:

Agnostic

Atheist

Muslim

Buddhist

Hindu

Practicing Catholic

Non-practicing Catholic

Evangelist

Jewish

Jehovah's Witness

Other

1. Origin/ethnicity:

North American

South American

English

European

Eastern countries

North Africans

Sub-Saharan Africa

Asians

Oceania

Gypsy ethnicity

1. Type of partner relationship:

Living together as a couple

Steady partner not living together

No steady partner

1. If you have a partner: How long have you been with your partner?

Less than 2 years

2-5 years

5-10 years

More than 10 years

1. In case of cohabitation as a couple: How long have you been living together?

Less than 2 years

2-5 years

5-10 years

More than 10 years

1. Do you have or have you had sexual intercourse with more than one person during the same period of time?

Yes

No

1. No. of CURRENT sexual partners
2. Caregivers at home (children/elderly people)):

Yes

No

1. Sexual orientation:

Homosexual

Bisexual

Straight

Other

1. How often do you have sex?

Daily

Weekly

Monthly

Sporadic

1. In your sexual relations do you use any method of contraception (including reverse... Ogino... condom...)?

Always

Most of the time

Sometimes

Never

1. What method of contraception do you use at the moment?

None

Natural methods (temperature, Ogino method, reverse gear, breastfeeding...)

Barrier methods (female condom, male condom, diaphragm, cap)

ACHO (classic combined pill)

Gestagen-only pill (GSA)

Patch

Ring

Quarterly injection

Implant

Copper IUD

LNG IUD

1. If you are already using one method of contraception, why would you want to change to another?

Problems with frequency of use

Side effects

Problems with sexual relations

Economic issues

Effectiveness

Pregnancy using the method

Other

QUESTIONS

1. What is your main motivation for having sex?

Love/intimacy

Pleasure

Procreation

Pleasing your partner

1. How well do you and your partner communicate about sexual matters?

Very well

Well

Normal

Poorly

Never

1. Are you worried that your partner might be sexually affected by (notice/disturbed by) your method of contraception?

Yes

No

Sometimes

1. Would a method that could negatively affect your sexual relations (desire, lubrication, orgasm, satisfaction) be an option for you?

Not at all

It could be considered

I would accept it

1. Would you find it interesting if the contraceptive method you choose is part of sexual play (the eroticism of sex)?

Yes

No

Don't know

1. Regarding your partner's relationship with the contraceptive method. What do you prefer:

Your partner is aware of its use and may feel it.

Knows of its use but does not notice it

Does not know of its existence and does not notice it

1. Are you worried about it being known that you are using a contraceptive method?

Yes

No

Sometimes

1. Would pre-sex use make you feel more confident during sex?

Yes

No

Don't know

1. Would you mind having to touch your own genitals to use the method?

Yes

No

Don't know

1. Would it be inconvenient for you to have to use a method that requires learning on your part?

Yes

No

Don't know

1. If the method you use requires proper positioning, would you abandon yourself to pleasure without thinking that you might have misused it at the time?

Yes

No

Don’t know

1. Does a method that requires daily use give you peace of mind during intercourse?

Yes

No

Don’t know

1. Does vaginal bleeding between periods .... interfere negatively with your sexual activity?

Yes

No

Sometimes

1. Have you stopped having sex because of your period?

Yes

No

Sometimes

1. If you answered yes/sometimes, did it bother you?

Yes

No

Sometimes

1. Do you prefer to have your period every month while using your method of contraception?

Yes

No

Don’t knowDon’t know

1. Do you resent relying on your partner to decide on protection during sex?

Yes

No

Don’t know

1. How do you value having sex spontaneously when it is your decision?

0 (Not important)---5 (Fairly important)---10 (Very important)

1. Do you have a sexual fantasy of getting pregnant?

Yes

No

Sometimes

1. Would you mind using a method that could reduce contact with your partner?

Yes

No

Sometimes

1. Do you think that the side effects of certain contraceptive methods can negatively affect your sex life?

Yes

No

Don’t know

1. Does it give you the same security (do you prefer) to touch/feel your method as not noticing it?

Yes

No

Don’t know

1. Would you feel sexually safe if you controlled your contraception by knowing your own body (temperature, possible ovulation days)?

Yes

No

Don’t know

1. Are you influenced by your partner regarding contraception?

Yes

No

Sometimes
